# Indole-3-acetic acid promotes differentiation while suppressing proliferation in human intestinal epithelial cells

**DOI:** 10.1007/s13577-026-01397-x

**Published:** 2026-06-12

**Authors:** Marta Mallardo, Furqan Memon, Raffaella Pagliaro, Aurora Daniele, Ersilia Nigro

**Affiliations:** 1 CEINGE-Biotecnologie Avanzate Scarl “Franco Salvatore”, Via G. Salvatore 486, 80145 Naples, Italy; 2 Dipartimento di Scienze dell’educazione e dello Sport, Centro Direzionale Isola F2, Università Telematica Pegaso, 80143 Naples, Italia; 3 Dipartimento di Scienze e Tecnologie Ambientali, Biologiche, Farmaceutiche, Università della Campania “Luigi Vanvitelli”, via Vivaldi 43, 81100 Caserta, Italy; 4 Dipartimento di Scienze Mediche Traslazionali, Università della Campania “L. Vanvitelli”, 80131 Naples, Italy; 5 U.O.C. Clinica Pneumologica L. Vanvitelli, A.O. dei Colli, Monaldi Hospital, 80131 Naples, Italy; 6 Dipartimento di Medicina Molecolare e Biotecnologie Mediche, Università degli Studi “Federico II”, via Pansini, 5, 80131 Naples, Italy

**Keywords:** Indole-3-acetic acid, Intestinal epithelial Caco-2 cell line, Epithelial differentiation, Cell viability

## Abstract

Indole-3-acetic acid (IAA) is a tryptophan-derived microbial metabolite increasingly recognized for its role in intestinal homeostasis, immune regulation, and epithelial function. However, the IAA molecular effects on intestinal epithelial cells remain incompletely defined. Here, we investigated the effects of increasing IAA concentrations on intestinal epithelial biology using intestinal epithelial Caco-2 cell line, with a focus on cytotoxicity, epithelial repair, and differentiation. Cell viability was assessed by MTT assay; colony-forming assays were used to evaluate stemness potential; cytokine’s expression was quantified by qPCR. Differentiation was analyzed through dome formation and analysis of DPPIV, SI, KLF4 and E-cadherin differentiation markers by qPCR and immunofluorescence. IAA treatment exerts concentration-dependent effects, reducing the viability and colony-forming capacity of Caco-2 cells. At higher concentrations, IAA also decreased IL-17, IL-1β, IL-6 expression. Interestingly, IAA enhanced dome formation and upregulated several differentiation markers, suggesting a shift toward a more mature epithelial phenotype. Altogether, our results indicate that IAA directly influences epithelial cell biology by modulating viability, inflammatory signaling, repair capacity, and differentiation. Our data support a potential role for this metabolite in regulating intestinal growth, repair and differentiation suggesting that IAA may contribute to intestinal homeostasis and disease pathophysiology. However, further studies are essential to understand the potential therapeutic implications related to colorectal cancer.

**Graphical abstract:**

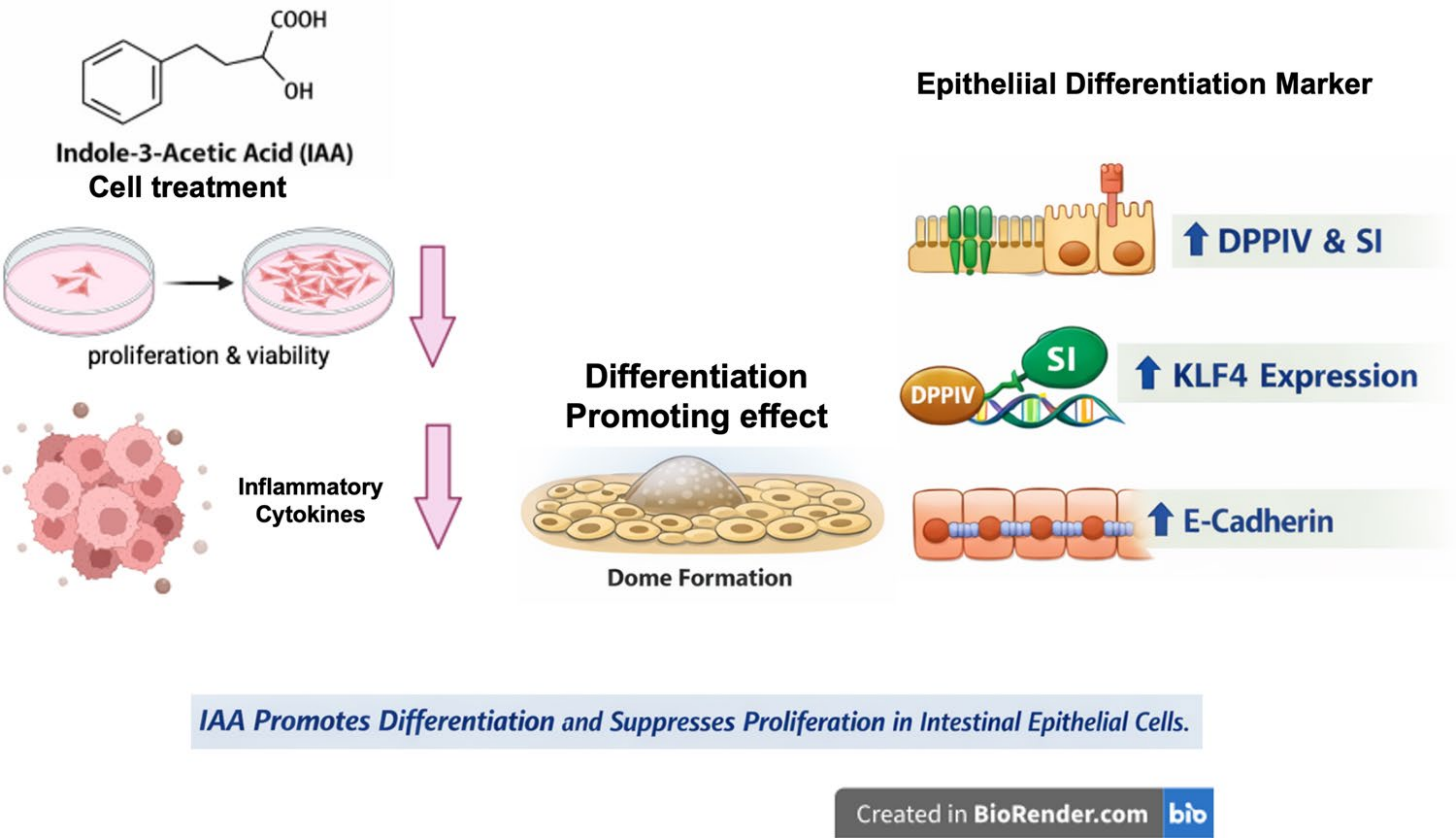

**Supplementary Information:**

The online version contains supplementary material available at 10.1007/s13577-026-01397-x.

## Introduction

Dietary habits strongly influence the production and bioavailability of indole-3-acetic acid (IAA), a tryptophan-derived microbial metabolite with emerging roles in intestinal and systemic health [1–3].

Among dietary components, tryptophan-rich foods such as legumes, dairy products, meat, fish, and cruciferous vegetables serve as precursors for microbial metabolites [4]. One of the most abundant and biologically active metabolites is IAA, produced by specific members of the intestinal microbiota [5]. Previous reports indicate that circulating IAA concentrations in human plasma are typically in the low micromolar range [6]. Additionally, fecal concentrations have been reported to be approximately ~ 5 µM [7]. Emerging evidence indicates that IAA exerts protective effects within the gastrointestinal tract, including the reinforcement of epithelial barrier integrity, anti-inflammatory and antioxidant activity, and immunomodulatory activities [8]. In fact, in the gastrointestinal tract, IAA contributes to epithelial integrity, maintenance of the intestinal barrier, and suppression of inflammation [9, 10], making it a promising candidate for the management of gastrointestinal chronic disorders and even cancer [11]. On the other hand, recent studies further have suggested [1] that fluctuations in microbiota-derived indole metabolites may contribute to chronic intestinal disorders, including inflammatory bowel disease (IBD) and colorectal cancer (CRC) [1, 11]. Of note, patients with CRC have been reported to exhibit reduced circulating and fecal levels of IAA, suggesting a potential association between dysregulated tryptophan metabolism and tumorigenesis [12, 13]. Moreover, IAA has demonstrated context-dependent effects on cellular processes including oxidative stress regulation, autophagy, apoptosis, and epithelial barrier stability. Furthermore, IAA participates in the formation of innate and adaptive immunity [11, 14, 15] partly by modulating reactive oxygen species and autophagy [1, 16]. Also, we and others have demonstrated that IAA exhibits a toxicity against cancer such as neuroblastoma and prostate cancer cells [17–19]; the molecular mechanisms responsible for such anti-cancer activity are still under investigation and controversial although the control of cell cycle, autophagy, and apoptosis have been demonstrated. Recently, an IAA inhibition of the intestinal stem cell turnover, epithelial proliferation, and tumorigenesis in mouse models of CRC was reported [20].

Therefore, the effects of IAA on the intestinal epithelium are not yet fully characterized, e.g., on epithelial viability, inflammatory signaling, and differentiation. In this context, the Caco-2 human intestinal epithelial cell line provides a well-established model to explore these biological processes. Therefore, this study aimed to investigate the biological effects of IAA on Caco-2 cells by assessing: (1) cell viability and colony-forming capacity; (2) modulation of inflammatory cytokine expression, and effects on wound closure ability; (3) the capacity of IAA to regulate epithelial differentiation.

## Materials and methods

### Cell culture

The established intestinal epithelial Caco-2 cell line was kindly provided by the Bank of Human and Animal Continuous Cell Lines-CEINGE Biotecnologie Avanzate “Franco Salvatore”, Napoli, Italy. Cells were grown in Dulbecco’s modified Eagle’s medium (DMEM) (Invitrogen, MA, USA) supplemented with 20% heat-inactivated fetal bovine serum (FBS), 2 mM l-glutamine, penicillin (100 U/mL), and streptomycin (100 mg/mL) and kept in CO_2_ incubator with 5% CO_2_. All experiments were performed in complete medium with various concentrations of IAA (0.01, 0.1, and 1 mM). The IAA doses were selected based on published evidence [17].

### MTT cell viability assay

Cell viability was assessed using the 3-[4,5-dimethylthiazol-2-yl]-2,5-diphenyltetrazolium bromide (MTT) colorimetric assay, following the manufacturer’s instructions. Briefly, Caco-2 cells were seeded in 96-well plates at a density of 4 × 10^3^ cells per well and incubated overnight in DMEM supplemented with 20% FBS. The following day, cells were exposed to increasing concentrations of IAA (0.01, 0.1, and 1 mM), while control wells received medium alone. After 24, 48, and 72 h of treatment, MTT reagent was added and processed as previously described [21]. Experiment was performed three times in triplicate.

### Colony assay

Caco-2 cells were seeded in six-well plates at the concentration of 1 × 10^3^ cells/well. After 24 h, cells were treated with IAA (0.01, 0.1, and 1 mM) and allowed to grow for 7 days. The growth medium was replaced every 3 days. After 7-day incubation, these plates were washed with PBS twice, fixed by 4% paraformaldehyde (PFA) (Invitrogen, CA, USA) for 30 min and colored in 1% crystal violet solution for 20 min and washed repeatedly in water. Colonies were counted manually using a light microscope as previously described [22]. Experiments were performed three times in triplicate.

### Wound healing

Caco-2 cells were seeded in six-well plates at a density of 3 × 10^5^ cells/well in complete culture medium and allowed to grow to confluence. Then cells were treated with mitomycin (4 μg/mL; Sigma-Aldrich) for 2 h to inhibit proliferation and a linear wound was generated using a tip. Following PBS washes, cells were incubated with IAA (0.01, 0.1, and 1 mM), with untreated cells serving as controls. Wound closure was monitored by imaging the same positions along the scratch immediately after wounding (0 h) and at 24, 48, 72, and 96 h using an inverted phase-contrast microscope (Nikon TS100, equipped with fluorescence and video camera). The extent of wound closure was quantified using ImageJ software and expressed as the percentage of wound closure as previously described [17]. Experiments were performed three times in triplicate.

### Dome formation and analysis

Caco-2 cells were seeded at a density of 2 × 10^5^ cells/cm^2^ in 12-well plates (Corning Life Sciences, MA, USA) pre-coated with rat tail Type I collagen (BD Biosciences, CA, USA) and grown until confluence. The culture medium was replaced every 3 days. On day 7 after reaching confluence, cells were treated with increasing concentrations of IAA (0.01, 0.1, and 1 mM) for 48 h, with the drug refreshed every 24 h. Cells incubated in medium alone served as negative controls (NC). Dome formation was monitored using the Cell Discoverer 7 system through time-lapse image acquisition over a 48 h period, and the number of domes was quantified by counting at least 10 microscopic fields for each condition.

### RNA extraction and quantitative real-time PCR

Total RNA was extracted from Caco-2 cells by using TRIzol Reagent (Thermo Fisher Scientific, MA, USA) according to the manufacturer’s instruction. The RNA concentration was quantified by using fluorescence-based detection with Qubit 4 Fluorometer (Thermo Fisher Scientific, MA, USA). One microgram of total RNA was subjected to reverse transcription with SuperScript IV VILO Master Mix (Invitrogen, MA, USA) according to the manufacturer’s instructions. Gene expression analysis was carried out using the C1000 Touch Thermal Cycler (Bio-Rad, CA, USA) and PowerUp SYBR Green Master Mix (Applied Biosystems, MA, USA). The thermal cycling protocol was previously described [18]. GAPDH was used as the housekeeping gene, and fold changes were calculated using the 2^−ΔΔCt^ method, as previously reported [17]. Primer sequences used for qRT-PCR are shown in Table 1. Experiments was performed three times in duplicate.

**Table 1:** Primer sequences used for q-RT-PCR experiments

*Target genes*	*Primers (5' to 3')*
DPPIV	F: GGCACCTGGGAAGTCATCGGGA
	R: AGAGGGGCAGACCAGGACCG
SI	F: CATCCTACCATGTCAAGAGCCAG
	R: GCTTGTTAAGGTGGTCTGGTTTAAATT
KLF-4	F: ATCTTTCTCCACGTTCGCGTCTG
	R: AAGCACTGGGGGAAGTCGCTTC
E-cadherin	F: GGCCAGGAAATCACATCCTA
	R: GGCAGTGTCTCTCCAAATCC
IL-17	F: CGGACTGTGATGGTCAAC
	R: CAAGGTGAGGTGGATCGGTT
IL-1β	F: CCTGAGCTCGCCAGTGAAAT
	R: GTCGGAGATTCGTAGCTGGA
IL-6	F: CCAGAGCTGTGCAGATGAGT
	R: AAGTGGCATTGCATCCCTGA
GAPDH	F: AGCCACATCGCTCAGACAC
	R: GCCCAATACGACCAAATCC

### Western blotting

Total protein was extracted from cells using pre-cooled radioimmunoprecipitation assay (RIPA) buffer (Sigma-Aldrich, MO, USA) supplemented with a protease inhibitor cocktail (Abcam, Cambridge, UK). Protein concentrations were determined using the Bradford assay (Bio-Rad, CA, USA), and samples were prepared as previously described [23]. Briefly, thirty μg of total protein were separated by SDS-PAGE and transferred to PVDF membranes (Pierce Biotechnology, MA, USA). Subsequently, membranes were incubated overnight at 4 °C with primary antibodies against KLF-4, E-cadherin, and GAPDH (Cell Signaling Technology, MA, USA), followed by incubation with anti-rabbit antibody (Cell Signaling Technology, MA, USA). Finally, protein bands were visualized using the ChemiDoc XRS system (Bio-Rad, CA, USA) with ECL reagents (Pierce Biotechnology, MA, USA). Experiments were performed three times in duplicate.

### Immunofluorescence and image analysis

Cells were seeded on glass coverslips, fixed for 10 min at RT with 4% PFA (Invitrogen, CA, USA), washed with PBS (three washes of 5 min each), permeabilized with 0.1% Triton X-100 in PBS for 15 min at RT, and rinsed with PBS at RT. In order to evaluate differentiation markers, cells were pre-incubated for 30 min with normal goat serum (1:10) diluted in PBS at RT to saturate the non-specific binding sites. Then the cells were blocked using 0.1% Triton 100X and 5% FBS in PBS 1X for 1 h at room temperature. The cells were then incubated overnight at 4 °C with primary antibodies in 0.1% Triton 100X and 5% FBS in PBS 1X. The primary antibodies were KLF-4 and E-cadherin (Abcam CB, UK; Cell Signaling Technology, MA, USA). The following day, the cells were incubated at RT with secondary antibodies: Alexa Fluor ^®^ 488 goat and Alexa Fluor ^®^ 594 goat (Thermo Fisher Scientific Massachusetts, USA). Nuclei were counterstained with DAPI according to the manufacturer’s instruction (Thermo Fisher Scientific Massachusetts, USA). Experiments were performed two times in triplicate.

### Statistical analysis

Data are expressed as the means of replicates ± the standard deviation (SD). GraphPad Prism 6 software (GraphPad Software, CA, USA) was used to carry out the analyses. Statistical comparisons between the control and treatments were performed using the one-way or two-way ANOVA followed by the Tukey multiple comparisons test. A *p* value < 0.05 was considered statistically significant.

## Results

### IAA reduces the viability and colony-forming capacity of Caco-2 colorectal adenocarcinoma cells while reducing the expression of pro-inflammatory cytokines

**Fig. 1:**
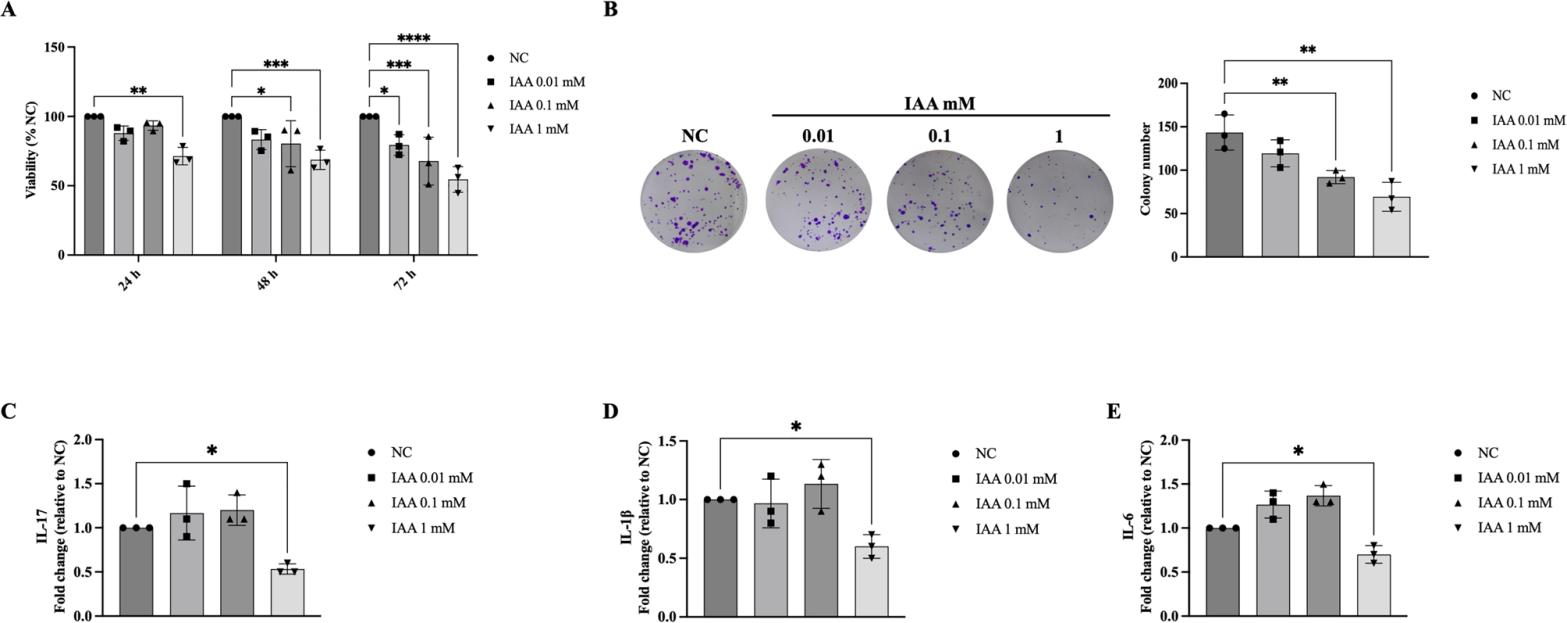
IAA reduces Caco-2 cell viability and colony formation in a time- and dose-dependent manner. **A** Cell viability of Caco-2 cells after 24, 48, and 72 h of treatment with IAA (0.01, 0.1, 1 mM) was assessed via MTT assay. **B** Caco-2 cells were exposed to IAA (0.01, 0.1, 1 mM) for 7 days and assessed for colony formation; representative images of colonies and their quantization are reported. (C, D, E) Caco-2 cells were exposed to IAA (0.01, 0.1, 1 mM) for 48 h and cytokine expression (IL-17, IL-1β, IL-6) was evaluated trough qPCR normalized on the housekeeping gene expression. Values are expressed as the mean ± SD of three independent experiments performed in triplicate. *p value < 0.05; **p value < 0.01; ***p value < 0.001. *NC* negative control, *IAA* indole-3-acetic acid

To evaluate the effects of IAA on cell viability, Caco-2 cells were treated with increasing concentrations of the molecule (0.01, 0.1, and 1 mM) for 24, 48, and 72 h, after which an MTT assay was performed. Our data show that IAA reduced Caco-2 cell metabolic activity consistent with anti-proliferative effects in a time- and dose-dependent manner (Fig. 1, panel A). Specifically, after 24 h, a significant reduction in cell metabolic activity consistent with anti-proliferative effects was observed only at the highest dose. At longer incubation times, all doses were effective in decreasing cell viability, with the 1 mM concentration showing the strongest effect at all time points. Consistently, a similar trend was observed in the colony formation assay (Fig. 1, panel B), as the number of colonies significantly decreased with increasing IAA concentrations. The expression of key cytokines involved in the control of the inflammatory response in the epithelial intestinal Caco-2 cells was investigated through qPCR (Fig. 1, panels C, D, E). In detail, the expression of IL-17, IL-1β, and IL-6 was evaluated upon treatment with IAA (0.01, 0.1, and 1 mM) for 48 h.

#### IAA progressively impairs Caco-2 cell migration

**Fig. 2:**
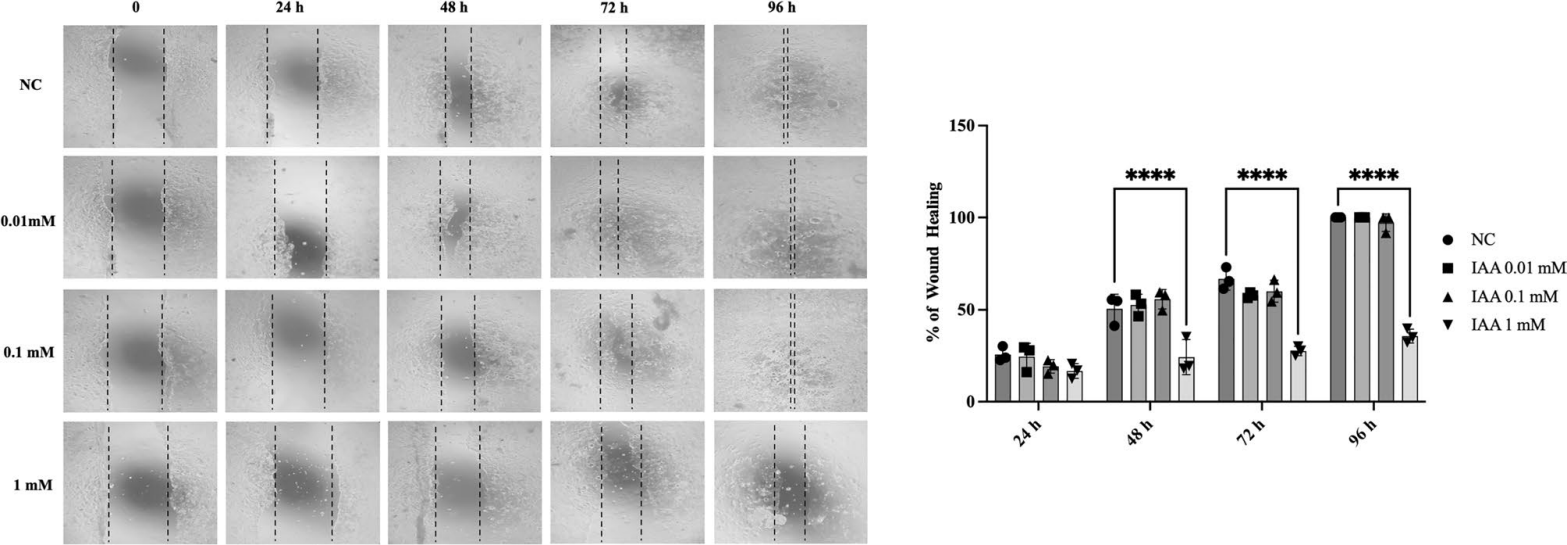
IAA impairs Caco-2 cell migration. Representative images of wound closure in NC and IAA-treated Caco-2 cells are shown in the left panel. Dashed lines indicate the initial wound boundaries. Low and intermediate IAA doses did not alter wound progression, whereas the highest dose markedly impaired closure, resulting in wider remaining gaps. Quantification of the residual wound width (right panel) confirmed a significant reduction in migratory capacity only at the highest IAA concentration. Values are expressed as the mean ± SD of three independent experiments performed in triplicate. *p value < 0.05; ****p value < 0.0001. *NC* negative control, *IAA* indole-3-acetic acid

To evaluate the migratory capacity of Caco-2 cells following exposure to IAA, we performed a wound-healing assay. The pre-treatment with mitomycin to inhibit proliferation guaranteed the evaluation of the linear wound closure rather than the proliferation. We found that at 0.01 and 0.1 mM IAA concentrations, the wound edges advanced at a rate comparable to the control, indicating that these doses did not substantially affect cell migration (Fig. 2). Interestingly, at 1 mM IAA concentration, cells showed marked reduced migration, with wound borders that advanced poorly and left a significantly central gap. For summarize, while the two lower IAA concentrations produced wound widths that were comparable to the control, the highest dose resulted in a significant increase in residual gap size, indicating a strong inhibitory effect on Caco-2 cell migration.

#### IAA promotes dome formation in Caco-2 cells

**Fig. 3:**
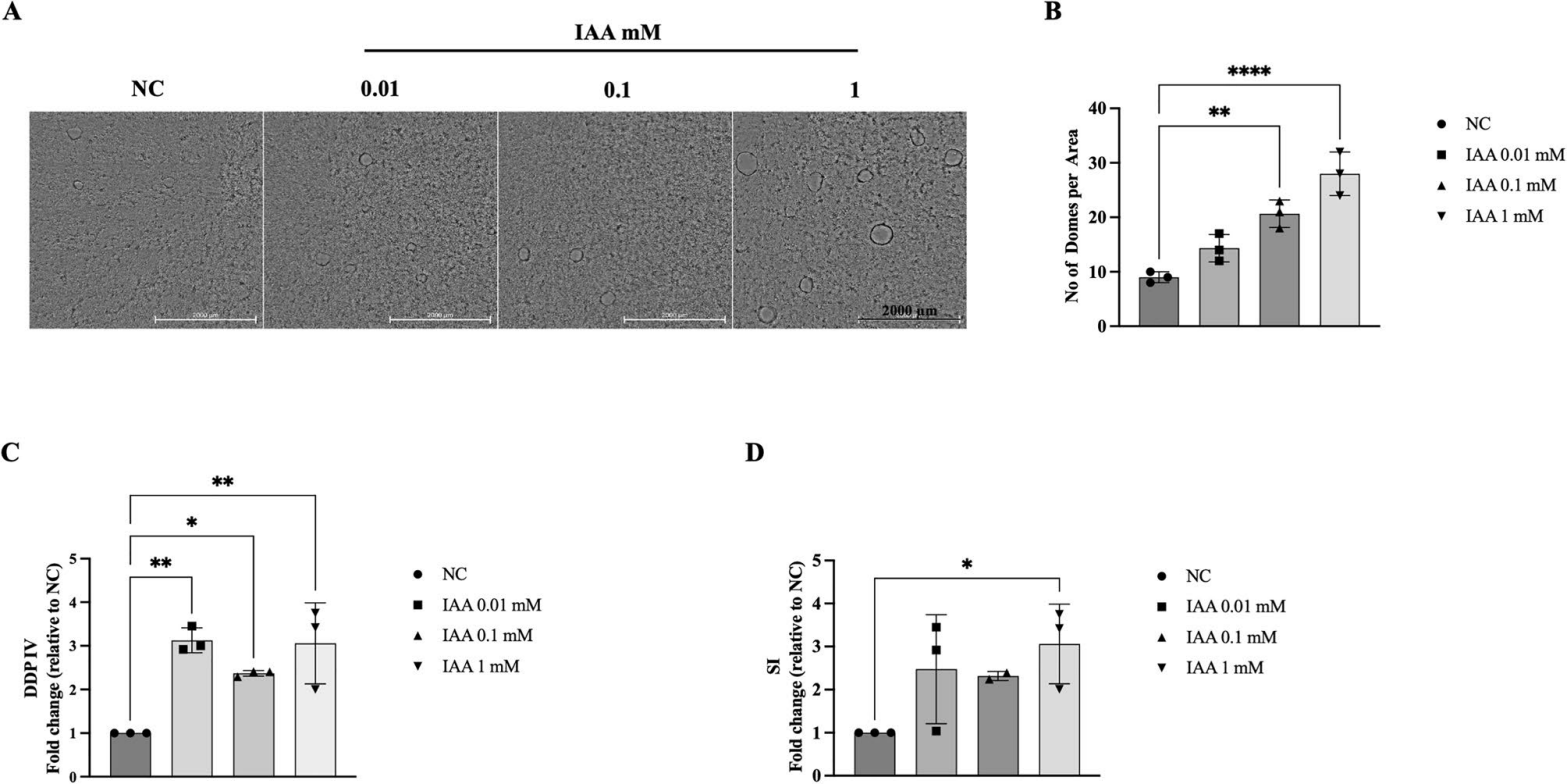
IAA induces a dose-dependent increase in dome formation and enhances intestinal differentiation markers DPP IV and SI at mRNA levels in Caco-2 cells. Caco-2 cells were treated with increasing doses of IAA (0.01, 0.1, 1.0 mM) for 48 h and assessed for domes formation. **A** Representative images of domes and (**B)** their quantification are shown. The mRNA expression levels of DPP IV (**C**) and SI (**D)** were assessed after 48 h of IAA treatment via qPCR. Data were normalized to GAPDH and analyzed using the 2^−ΔΔCt^ method. Values are expressed as the mean ± SD of three independent experiments performed in triplicate. Scale bar = 2000 μM. *p value < 0.05; ****p value < 0.0001. *NC* negative control, *IAA* indole-3-acetic acid

We next investigated whether IAA treatment affected the differentiation capacity of Caco-2 cells by evaluating dome formation, as well as the expression of differentiation markers such as dipeptidyl peptidase-4 (DPP IV) and Sucrase-Isomaltase (SI). As shown in Fig. 3, panels A and B, IAA treatment induced a dose-dependent increase in dome formation, reaching up to a 2.9-fold (approximately threefold) increase at the highest IAA dose. This marked increase in domes, a well-established functional marker of enterocyte polarization and maturation, indicates that IAA effectively promotes intestinal epithelial differentiation.

#### IAA modulates differentiation marker expression in Caco-2 cells

To further explore the involvement of IAA in the differentiation process, we assessed the mRNA expression levels of the brush border enzymes DPP IV and SI (Fig. 3, panels C and D). Compared to untreated control, IAA-treated cells exhibited an approximately threefold increase in DPP IV expression, already evident at the lowest dose tested (Fig. 3, panel C). Similarly, SI mRNA levels were elevated following IAA treatment across all doses, although statistical significance was reached only at the highest concentration (Fig. 3, panel D). The upregulation of both markers strongly supports the hypothesis that IAA promotes differentiation of Caco-2 cells.

**Fig. 4:**
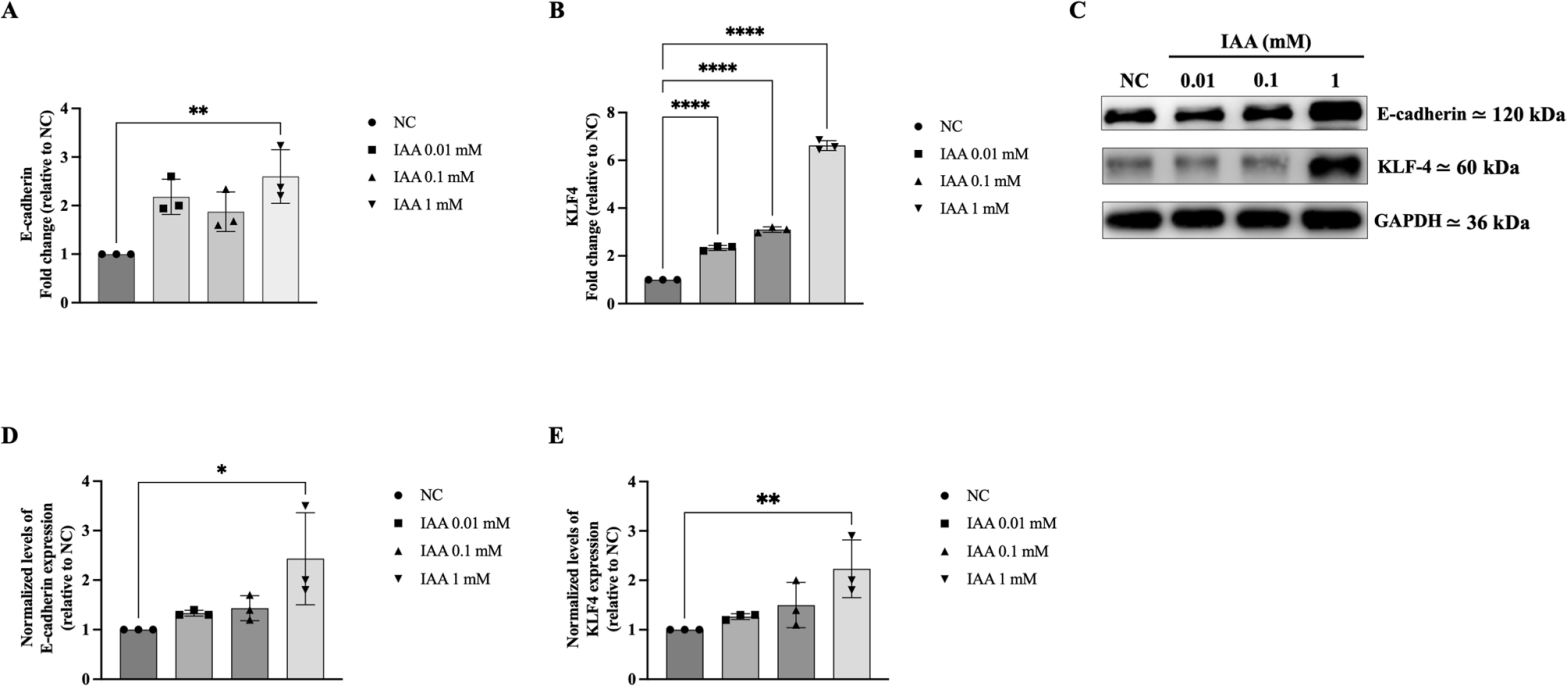
IAA induces a dose-dependent increase in KFL4 and E-cadherin expression at both the mRNA and protein levels in Caco-2 cells. **A**, **B** mRNA expression levels of KLF-4 and E-cadherin were evaluated after 48 h of IAA treatment in Caco-2 cells using qPCR. Data were normalized to GAPDH and analyzed using the 2^−ΔΔCt^ method. **C**–**E** Representative Western blot images of KLF-4 and E-cadherin after 48 h of IAA treatment, together with the corresponding densitometric quantifications, are shown. GAPDH was used as the loading control. Values are expressed as the mean ± SD of three independent experiments performed in triplicate. *p value < 0.05; **p value < 0.01; ****p value < 0.0001. *NC* negative control, *IAA* indole-3-acetic acid

**Fig. 5:**
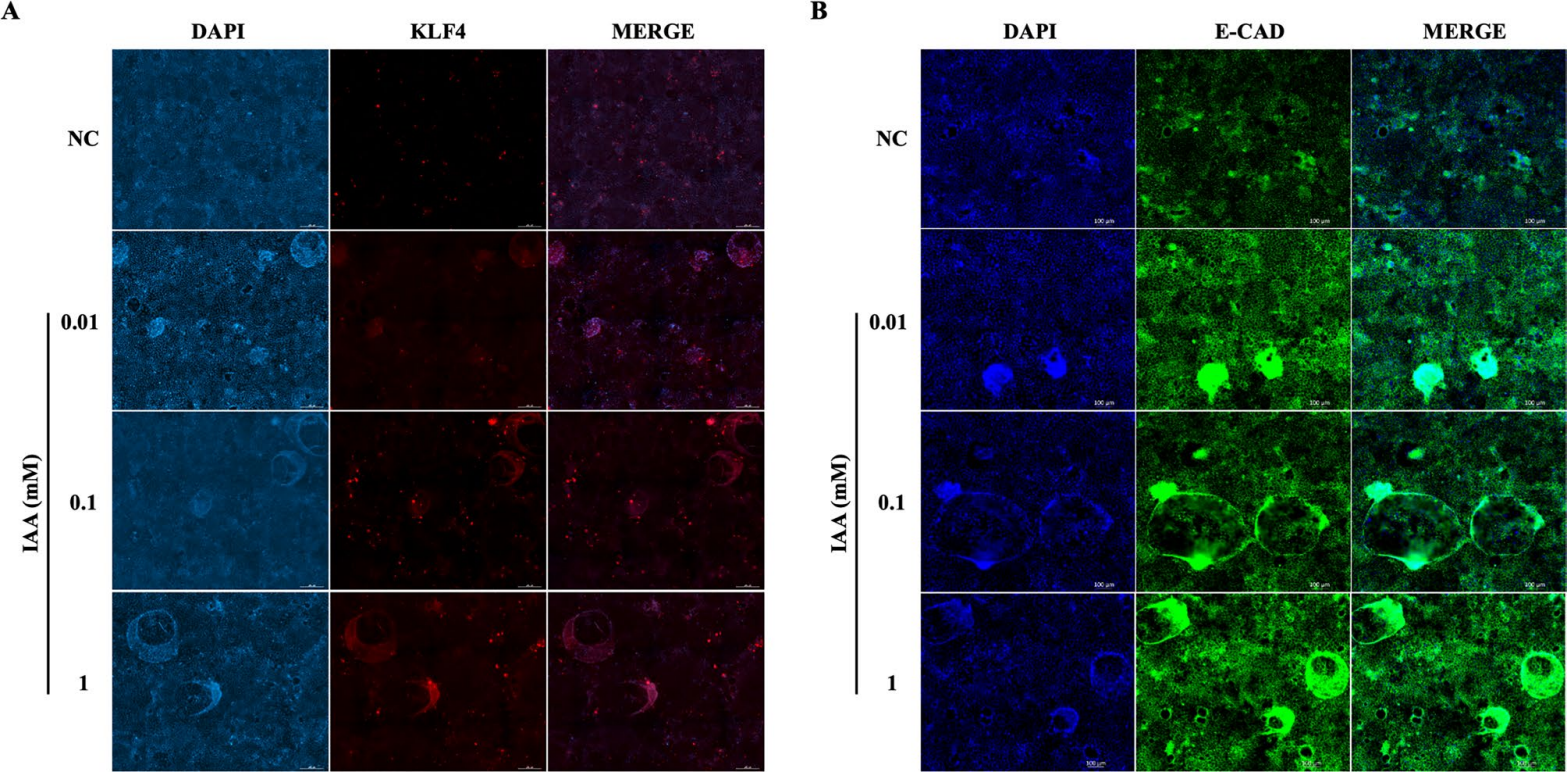
Immunofluorescence staining of KLF-4 and E-cadherin expression in Caco-2 cells. Immunofluorescence staining of KLF-4 (**A**) and E-cadherin (**B**); cells were incubated with anti-KLF-4 and E-cadherin antibody and then counterstained with DAPI to label the nuclei. Magnification of both images is shown in supplementary 1. *NC* negative control, *IAA* indole-3-acetic acid. Scale bar = 100 μm

Given the pivotal roles of KLF-4 and E-cadherin in regulating cellular differentiation and adhesion, we next investigated whether their expression was modulated by IAA administration. Our results revealed that a 48 h IAA exposure induced a significant upregulation of KLF-4 at both the mRNA and protein levels (Fig. 4, panels A and B). Notably, treatment with the highest dose of IAA led to a sixfold increase in KLF-4 mRNA expression compared to control (Fig. 4, panel A). Similarly, E-cadherin expression was increased at both the transcriptional and protein levels, with the strongest effect observed at the highest IAA dose (Fig. 4, panels C and D). The upregulation of both proteins was further confirmed by immunofluorescence analysis (Fig. 5). Under the highly confluent conditions required for differentiation, Caco-2 cells appear as a dense, polarized epithelial layer, with nuclei located at different focal planes. To improve image clarity, a higher magnification of a representative merged area is shown in Supplementary Fig. 1, allowing better visualization of the DAPI and KLF-4/E-cadherin signals.

## Discussion

This study demonstrates that IAA influences multiple aspects of Caco-2 biology, including viability, inflammatory signaling, and differentiation confirming that IAA may be considered as a potential mediator connecting nutrition, cell proliferation, and intestinal cancer risk.

Diet and microbial metabolism play a fundamental role in regulating intestinal physiology and modulating the risk of colorectal cancer (CRC). Among the natural indole metabolites, indole-3-acetic acid (IAA), particularly abundant and biologically active, has attracted attention for its immunomodulatory and protective properties on the intestinal barrier and thus as a potential mediator of epithelial homeostasis [24].

IAA in fact modulates several mammalian cellular functions, including oxidative stress responses, proliferation, and apoptosis [17]. In murine models, IAA has been shown to exert potent anti-inflammatory effects [11]. Additionally, IAA displays cytotoxic activity against human cancer neuroblastoma cell lines [17], melanoma cells [18], and PC-3 prostate carcinoma [19], although the underlying mechanisms remain incompletely understood.

The results of this study indicate that IAA, in a dose-dependent manner, reduces the viability of Caco-2 cells and their ability to form colonies, consistent with data showing that indole metabolites can exert anti-proliferative effects in various types of tumor cells [25]. In particular, cytotoxic effects emerge especially at the highest concentrations tested. In line with our results, IAA suppresses cell proliferation of various cellular type [17, 19, 25]. Specifically, regarding the Caco-2 cells, only one publication reported cytotoxic effects of IAA while no effects have been described for HepG2 hepatocellular cells [26]. On the other side, to our knowledge, there are no data reporting an increase of viability following IAA incubation. Regarding the colony formation ability, we previously demonstrated the ability of IAA to reduce colony in neuroblastoma cell lines [14], but no additional data are available in different cell models. Our evaluation of IAA effects in wound repair clearly showed an inhibitory effect of the molecule; to our knowledge, such activity has never been assessed but data on different indole molecules are available. Lee et al. found that indole-3-carbinol suppressed the proliferation of human colorectal carcinoma cell line (LoVo) [27].

Another finding of our study concerns the expression of key cytokines involved in controlling the inflammatory response: IAA treatment reduces the expression of the pro-inflammatory cytokines IL-17, IL-1β, and IL-6 by Caco-2 cells. These results are consistent with published evidence demonstrating that microbial indoles attenuate inflammatory signaling in epithelial and immune models, particularly pro-inflammatory cytokines (e.g., TNF-α, IL-6, IL-1β), and/or inhibit the NF-κB/TLR4 pathways in Caco-2 or closely related intestinal cell models [28, 29]. These results support the possibility that IAA contributes to a better mucosal environment. However, cytokine changes were measured only at mRNA level, and further protein-level studies are needed to confirm the effective functional immunomodulation.

Another interesting finding from this study is that, following exposure to IAA, Caco-2 cells exhibited increased dome formation (up to 2.9-fold at the maximum IAA dose of 1.0 mM) and an upregulation of the differentiation markers; in particular, we focused on DPP IV and SI at mRNA levels and on KFL4 and E-cadherin at both mRNA and protein levels. These results suggest that IAA promotes epithelial maturation and appears to strengthen barrier properties. To our knowledge, there is no evidence of a role for IAA in controlling colon cells differentiation. However, a recent review states that IAA suppressed β-catenin signaling in the colon of mice, suggesting a link between indole metabolites and intestinal stem cell (ISC) proliferation/renewal [30]. It is also possible that, by increasing dome formation and reducing inflammation, IAA may create a more suitable microenvironment for normal epithelial cell differentiation. However, because the effects are more pronounced at higher concentrations than those reported in physiological settings, further studies are needed to clarify whether IAA exposure actively induces differentiation or whether the reduced proliferation selectively enriches already differentiated cells.

The apparent different cellular effects we found in monolayer *vs* domes structure, i.e., IAA suppress proliferation and growth of 2D Caco-2 cells while enhancing the differentiation model, may be due to the effect of IAA in accelerating the post-confluent differentiation program. In Caco-2 monolayers, dome formation is a hallmark of advanced epithelial polarization and vectorial ion/fluid transport. Reduced or slower proliferation is compatible with increased dome formation since IAA accelerates post-confluent differentiation and junctional maturation.

This study has several limitations that should be acknowledged. First, our findings are based on a single transformed intestinal epithelial cell line (Caco-2), which may not fully recapitulate the complexity of the native intestinal epithelium. In preliminary experiments performed in HCT-15 cells, IAA did not induce significant effects on cell viability under our experimental conditions, suggesting that its biological activity may be model-dependent. Therefore, future studies in additional intestinal epithelial cell lines and organoids are needed to better define the relevance and context-specific effects of IAA. Second, a possible caveat of this study is related to the intrinsic property of Caco-2 cells to spontaneously differentiate upon reaching confluence. Since IAA treatment reduces cell proliferation and viability, we cannot completely exclude a contribution of reduced proliferation to the observed increase in dome formation and differentiation markers. Furthermore, it is also worth noting that although mitomycin C was used to block cell proliferation before the wound-healing assay, at the highest IAA concentration tested, cells also showed reduced viability; thus, we cannot completely rule out that the impaired wound closure observed at this dose may partly reflect cytotoxic effects, in addition to a direct inhibition of cell migration The present study is a phenotypic and exploratory work, then additional mechanistic studies will be important to better define the pathways underlying these effects. Indeed, IAA has been reported to act as a ligand of the aryl hydrocarbon receptor (AhR), which is known to regulate several cellular processes, including inflammation, metabolism, and cell migration [31]. Although the involvement of AhR signaling was not directly investigated in the present study, it is plausible that the observed effects of IAA may be at least partially mediated through AhR-dependent pathways.

In conclusion, our data demonstrated that IAA modulates selected epithelial phenotypes in vitro. The modulation of viability, migration, and differentiation in Caco-2 cells suggests that IAA may contribute to the regulation of epithelial turnover and barrier maintenance in vivo. Furthermore, the observed changes in dome formation and differentiation markers suggest a role of IAA in shaping epithelial maturation and function. Together, these findings provide molecular insight into how indole derivatives may influence intestinal homeostasis, although the degree to which these findings translate to in vivo physiology remains uncertain. Further studies, including in vivo validation, are essential before proposing potential relevance for epithelial homeostasis and tumor-related processes.

## Supplementary Information

Below is the link to the electronic supplementary material.

Supplementary file1 (PDF 4802 KB)

## Data Availability

Data are avialable on request.
