# Supplementary figures and images for: Indole-3-acetic acid promotes differentiation while suppressing proliferation in human intestinal epithelial cells
Source: Hum Cell. 2026 Jun 12;39(6):87. doi: 10.1007/s13577-026-01397-x (PMC13263299; doi:10.1007/s13577-026-01397-x)

**A**

**1 mM**

**DAPI**

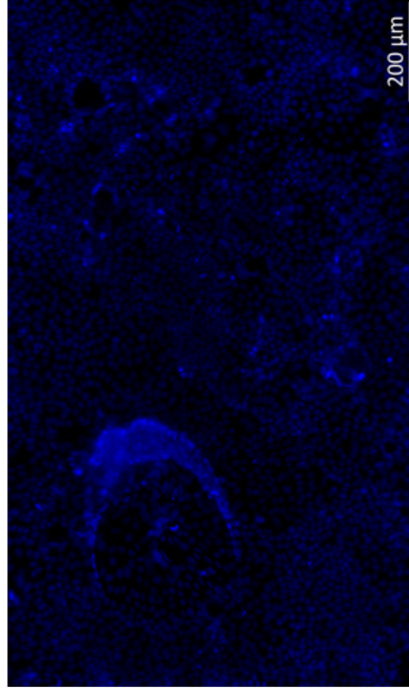

**KLF-4**

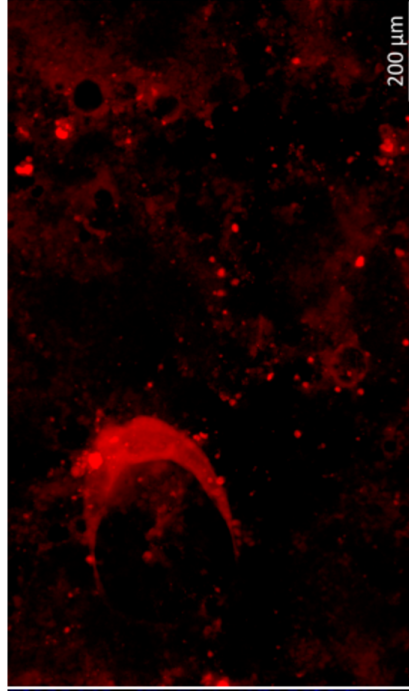

**MERGE**

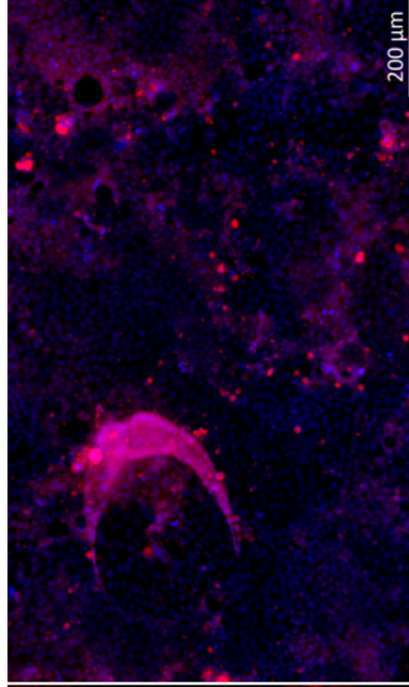

**B**

**1 mM**

**DAPI**

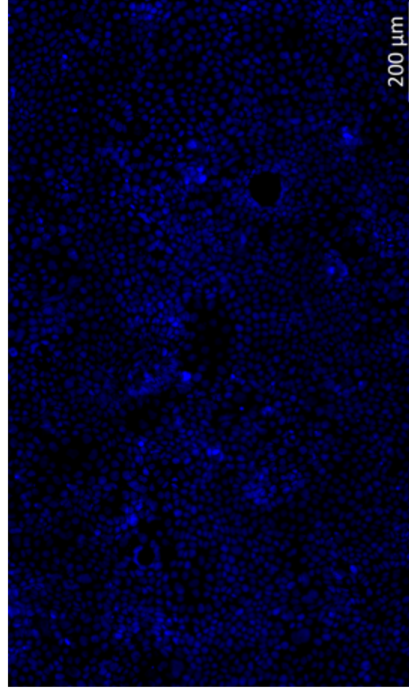

**E-cadherin**

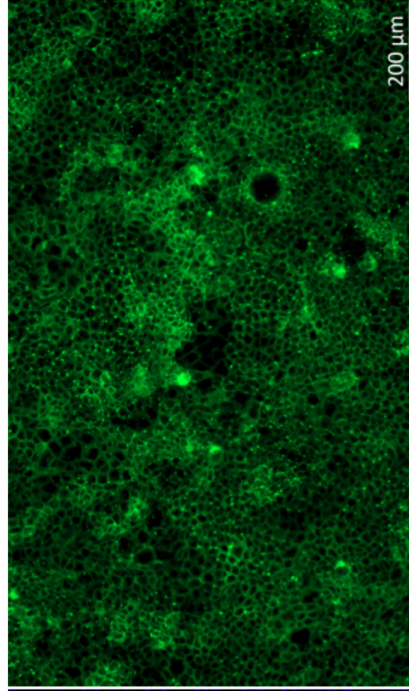

**MERGE**

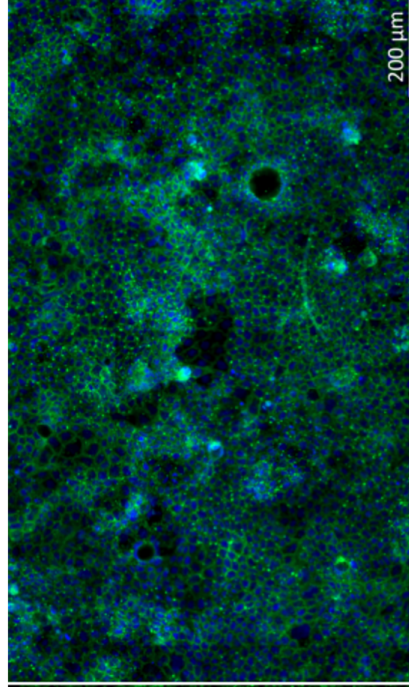

Supplement: Supplementary file 1 — Supplementary file1 (PDF 4802 KB) [file 13577_2026_1397_MOESM1_ESM.pdf]
